# The Impact of Pandemic Influenza H1N1 on Health-Related Quality of Life: A Prospective Population-Based Study

**DOI:** 10.1371/journal.pone.0017030

**Published:** 2011-03-02

**Authors:** Albert Jan van Hoek, Anthony Underwood, Mark Jit, Elizabeth Miller, W. John Edmunds

**Affiliations:** 1 Immunisation, Hepatitis and Blood Safety Department, Health Protection Agency, London, United Kingdom; 2 Applied Laboratory and Bioinformatics Unit, Health Protection Agency, London, United Kingdom; 3 Modelling and Economics Unit, Health Protection Agency, London, United Kingdom; 4 Centre for the Mathematical Modelling of Infectious Diseases, London School of Hygiene and Tropical Medicine, London, United Kingdom; University of Hong Kong, Hong Kong

## Abstract

**Background:**

While the H1N1v influenza pandemic in 2009 was clinically mild, with a low case-fatality rate, the overall disease burden measured in quality-adjusted life years (QALY) lost has not been estimated. Such a measure would allow comparison with other diseases and assessment of the cost-effectiveness of pandemic control measures.

**Methods and Findings:**

Cases of H1N1v confirmed by polymerase chain reaction (PCR) and PCR negative cases with similar influenza-like illness (ILI controls) in 7 regions of England were sent two questionnaires, one within a week of symptom onset and one two weeks later, requesting information on duration of illness, work loss and antiviral use together with EQ-5D questionnaires. Results were compared with those for seasonal influenza from a systematic literature review. A total QALY loss for the 2009 pandemic in England was calculated based on the estimated total clinical cases and reported deaths. A total of 655 questionnaires were sent and 296 (45%) returned. Symptoms and average illness duration were similar between confirmed cases and ILI controls (8.8 days and 8.7 days respectively). Days off work were greater for cases than ILI controls (7.3 and 4.9 days respectively, p = 0.003). The quality-adjusted life days lost was 2.92 for confirmed cases and 2.74 for ILI controls, with a reduction in QALY loss after prompt use of antivirals in confirmed cases. The overall QALY loss in the pandemic was estimated at 28,126 QALYs (22,267 discounted) of which 40% was due to deaths (24% with discounting).

**Conclusion:**

Given the global public health significance of influenza, it is remarkable that no previous prospective study of the QALY loss of influenza using standardised and well validated methods has been performed. Although the QALY loss was minor for individual patients, the estimated total burden of influenza over the pandemic was substantial when compared to other infectious diseases.

## Introduction

Influenza severity is usually characterised by the case-fatality rate (CFR). There are major problems with this measure as the denominator (the number of cases) is difficult to ascertain, resulting in widely varying estimates for the same viral strain [1]. Using the CFR to characterise severity ignores the burden of disease in the vast majority of individuals who have symptomatic influenza (possibly severe) but do not die. Many millions of individuals were infected with the pandemic strain of influenza A/H1N1v in 2009, and it is likely that many more will be infected by related strains in the coming years. In order to help evaluate the overall impact of the 2009 H1N1v pandemic on the health of populations it is necessary to measure the burden associated with non-fatal as well as fatal cases. One simple way to measure the impact would be to use a measure that combines morbidity and mortality in a single unit. Quality Adjusted Life Years (QALYs) are a commonly used metric that has this property. The EQ-5D is a generic preference-based instrument designed to measure the health related quality of life (QoL or QALY-weight) of any disease state. Using this instrument allows quantification of the severity of H1N1v on a comparable and standardised scale. It enables rational decisions to be made about interventions in future waves of H1N1v by comparing, for instance, the cost per QALY gained from such interventions with nationally accepted norms. In addition, it gives more in depth understanding of the impact of influenza on different aspects of well being.

The health-related quality of life detriment from a population-based sample of confirmed H1N1v patients was prospectively measured and compared to controls who were investigated because they had influenza like illness (ILI), but were not laboratory confirmed as H1N1v. The aims were: 1) to quantify the burden of H1N1v for individual patients and investigate factors, such as age and treatment with antivirals, that may affect this; 2) compare the severity of the 2009 strain to other infections that cause ILI and previous estimates of the severity of influenza from a systematic literature review; and 3) to estimate the overall burden attributed to H1N1v in the population. The findings can then be used to inform effectiveness and cost-effectiveness analyses on policy decisions related to the control of future waves of this or related viruses.

## Methods

### Prospective study of severity of H1N1v

The EQ-5D is a combination of a questionnaire and a valuation technique. The tool values health-related quality of life in five dimensions: mobility, self-care, usual activities, pain/discomfort and anxiety/depression. For each dimension there are three levels: no problems, some problems and severe problems. The overall health status is also measured using the visual analogue scale (VAS). The power of the EQ-5D is that it makes it possible to convert an outcome for each dimension of this scale into a quality of life score. It is recommended by the National Institute for Clinical Excellence for use in cost-effectiveness analyses in the UK [2]. During the early stages of the 2009 pandemic PCR confirmed cases of influenza A/H1N1v and a control group of PCR negative cases of ILI were identified. The PCR test used was validated and has a good specificity and a sensitivity of 95.4% [3]. During this time (weeks 27/28 2009) the containment phase of the response to the pandemic was still in place in England and all cases of influenza were being actively traced and centrally registered on a single database (Fluzone), irrespective of risk status, age group, complications, etc. Demographic, clinical, and epidemiological information was recorded on each case, including name, age, address, date of onset, and whether the case had been confirmed as H1N1v, tested and confirmed as not being H1N1v (discarded), or was awaiting test confirmation. The database was updated daily. Cases found to be negative for H1N1v (ILI controls) were not investigated further, and so their aetiology is unknown. From this database, patients who had confirmed H1N1v and those who had ILI but had tested negative for H1N1v, who had a date of onset within 1 week of the (then) current date were contacted by post and asked to take part in the survey. During the period of the study, two regions of England (London and the West Midlands) stopped investigating every case. To avoid biasing the results of the survey, we excluded cases from these regions.

The Fluzone database was checked daily during the recruitment period (weeks 27 and 28 2009) for new cases of ILI with recent onset (i.e. onset within 1 week of the day on which the database was checked) who were not resident in London or West Midlands. These were then contacted and asked to participate. The covering letter explained the study and contained instructions for completing the survey. The questionnaire asked for age, sex, presence of pre-disposing conditions (diabetes, asthma or other chronic respiratory diseases, chronic heart, kidney or liver disease, long-term neurological disease, or immuno-suppression), attendance at hospital, date of onset of symptoms, whether antivirals were being taken, and if so when they were first taken, and a checklist documenting their symptoms on the day of the survey and on their worst day of illness. In addition, they were asked to fill in the two copies of the EQ-5D, one for the worst day of their illness and one for the day they filled in the questionnaire. A second questionnaire was sent out two weeks after the first, which requested information on the total duration of symptoms, and absenteeism from work or school. Respondents were also asked to fill in another EQ-5D questionnaire on that day to obtain a baseline score for their health-related quality of life. In case there was no response from the first mailing a reminder was sent out, containing both questionnaires. Non-responders to the second questionnaire were not followed up. Patients could fill in the questionnaires by post or on-line (they were provided with a secure login to enable this).

Children (<16 years) were sent a child version of the EQ-5D [4] and questions were altered somewhat (e.g. absence from school instead of work). A separate question on the work loss of the parents due to disease in the child was added. In the covering letter (addressed to the guardian) it was suggested that older children fill in the survey themselves (with the assistance of the parent/guardian) and that for younger ones the parent/guardian fill out the survey on their behalf. Copies of the questionnaires and cover letters are available from the authors on request.

Enquiry to the NHS Research Ethics Committee indicated that ethics approval for this study was not required, since collection of QoL information from patients is part of the routine surveillance activities of the Health Protection Agency (HPA).

Only individuals with an ILI should have been investigated for H1N1v but to be certain, we asked respondents whether they had fever plus at least one other respiratory symptom on their worst day of illness. In the statistical analysis, only cases and control participants who recorded that they had symptoms consistent with an ILI were included. Differences between the two groups (confirmed cases and ILI controls) were tested having corrected for multiple comparisons using the Šidák correction (an exact version of the Bonferroni correction). For the QALY analysis, we only included patients for whom a complete set of data was available to calculate the QALY loss; this is an onset and end date, as well as quality of life weights for the worst day and the date of onset. The overall QALY loss was estimated to be the area denoted by the triangle with vertices being the background quality of life weight at onset date, the quality of life weight at the worst day and the time since onset of the worst day, and the background quality of life weight at the recovery date. Attribution of risk factors to the QALY score was investigated by linear regression. In the regression QALY scores were logged to take account of the skew in the original data. Statistical analysis was performed with R version 2.11.0.

### Systematic literature review

To compare our results with previous estimates of the quality of life detriment due to influenza we performed a literature review. PubMed was searched for the terms ‘influenza’ and ‘quality-adjusted life year’, ‘QALY’, ‘QALD’ or ‘EQ-5D’. The abstracts of all identified papers were reviewed, and original articles (not reviews) published in English were retained.

### Overall disease burden

To estimate the overall disease burden in England for the 2009 H1N1v pandemic, we focussed on the number of cases presenting with fever and those who died. The estimated number of people presenting with ILI (fever+respiratory symptom) was based on the estimated number of infections. To obtain the latter the estimated total number of clinical cases [5] in the first and second waves in England was multiplied by a factor 10. This factor is based on a comparison of the estimated clinical cases and seroprevalence after the first wave in England [6]. Although it might be justified to use a higher multiplication factor for the second wave based on mortality and other surveillance data [5], [7], the same multiplier was used for the whole period and can therefore be seen as a conservative approach. To obtain the estimated number of infected persons presenting with ILI, the number of infections was multiplied by the proportion of infections presenting with fever (27%) as estimated from an intensive household follow up during the initial stages of the 2009 pandemic [8]. The total burden expressed in QALYs was a multiplication of the QALY loss obtained in this study by the number of infections presenting with ILI, plus the QALY loss for fatal cases. The QALY loss for fatal cases was estimated as the average life-expectancy corrected for the expected quality of life in those years [9]. This assumes that each recorded death was actually caused by H1N1v, that there was no under-reporting of deaths, and that despite most deaths being in risk groups, the average life-expectancy was lost per death. The baseline estimate assumed no discounting of future life-expectancy. Discounting at 3.5% [2] was also used in the sensitivity analysis.

## Results

### Prospective study of severity of H1N1v

A total of 655 patients met the inclusion criteria and were sent a questionnaire, of whom 390 were confirmed cases and 265 were ILI controls. We received 287 responses, of which 269 reported ILI and were included in the analysis, 186 from confirmed cases and 83 from ILI controls. The response rate was significantly higher in the confirmed H1N1v group (48% vs 31% p<0.001). This difference was slightly larger in children (55% vs 31%).

The demographic composition of the two groups was similar (Table 1). Although there was a slightly higher fraction of the control group that was in a risk group (25% vs 19%) this was not significant. The hospitalisation rate was 8–9% in both groups. This high level of hospitalisation may represent heightened concern at the outset of the epidemic. Antiviral use was higher among the confirmed cases (although this was not significant after adjusting for multiple comparisons). The proportion of cases receiving antivirals within 2 days of onset was similar between the two groups.

**Table 1:** Background characteristic of patients.

	*Confirmed H1N1v ILI cases*	*ILI controls (non-H1N1v cases)*
IlI (fever+1 other symptom)	186 (96%)	83 (89%)
*Of those with ILI*		
Adults	115 (62%)	58 (70%)
Children	71 (38%)	25 (30%)
Risk group	36 (19%)	21 (25%)
Hospital admission	16 (9%)	7 (8%)
Antivirals	132 (71%)	44 (53%) p = 0.0065*
Antivirals within 2 days after onset	65 (35%)	26 (31%)

*not significant when corrected for multiple comparisons.

The symptoms recorded by both groups were similar (Table 2). The only significant difference was that the confirmed H1N1v cases recorded more occurrences of cough (90% vs 64%, p<0.001). The duration of symptoms was not known for everybody due to non-respondents to the second questionnaire. Nevertheless, the duration was similar for the two groups (average duration of 8.8 and 8.7 days respectively for the confirmed and control group). The duration of time off work was 7.3 days for the confirmed cases and 4.9 for the ILI controls: a significant difference using the Welch two sided t-test (p = 0.003). The worst day of disease appeared shortly after onset of the symptoms for both groups, however for the control group the worst day was slightly later (median 2 days) after onset than for the confirmed cases (median 1 day after onset) (Table 2).

**Table 2:** Symptoms reported by patients.

*Symptoms*	*Confirmed H1N1v ILI cases*	*ILI controls (non- H1N1v cases)*
Sore throat	152 (82%)	68 (82%)
Cough	167 (90%)	53 (64%) p>0.001
Headache	160 (86%)	69 (83%)
Tiredness	176 (95%)	77 (93%)
Chills	142 (76%)	49 (59%) p = 0.006*
Loss of appetite	147 (79%)	62 (75%)
Muscle pain	128 (69%)	54 (65%)
Joint pain	99 (53%)	51 (61%)
Nausea	87 (47%)	38 (46%)
Diarrhoea	46 (25%)	28 (34%)
Conjunctivitis	53 (28%)	18 (22%)
Average duration of symptoms (min-max)	8.8 (1–28) n = 133	8.7 (2–32) n = 56
Worst day (median, mean, modus)	1, 1.64, day 1	2, 2.18, day 1
Time off work information available	82 (44%)	39 (47%)
Average time off work (min-max)	7.3 (1–28)	4.9 (1–21) p = 0.003

*not significant when corrected for multiple comparisons.

All five of the dimensions measured in the EQ-5D were affected by ILI, in both groups of patients, though usual activities and pain/discomfort were the most affected (Table 3). Only about 5% of patients said that they had no problems with pain or discomfort on the worst day of illness, and 2% (8%) said they had no problems with usual activities on the worst day of their illness in the confirmed (control) groups.

**Table 3:** Impact on the 5 dimensions as measured in the EQ-5D.

	*No problems*		*Some problems*		*Severe problems*	
	H1N1v	ILI controls	H1N1v	ILI controls	H1N1v	ILI controls
***Background***						
**Self care**	**125 (98%)**	**51 (96%)**	**1 (1%)**	**2 (4%)**	**1 (1%)**	**0 (0%)**
**Mobility**	**122 (96%)**	**52 (98%)**	**4 (3%)**	**1 (2%)**	**1 (1%)**	**0 (0%)**
**Usual activities**	**115 (90%)**	**50(94%)**	**11(9%)**	**3 (6%)**	**1 (1%)**	**0 (0%)**
**Pain/discomfort**	**118 (93%)**	**50(94%)**	**8 (6%)**	**3 (6%)**	**1 (1%)**	**0 (0%)**
**Anxiety/Depression**	**123 (97%)**	**50(94%)**	**4 (3%)**	**3 (6%)**	**0 (0%)**	**0 (0%)**
***Worst day***						
**Self care**	**83 (46%)**	**38 (48%)**	**57 (31%)**	**28(35%)**	**41(23%)**	**13(16%)**
**Mobility**	**31(17%)**	**17 (20%)**	**72 (39%)**	**34 (41%)**	**81 (44%)**	**32 (39%)**
**Usual activities**	**3 (2%)**	**7 (8%)**	**53 (29%)**	**25 (30%)**	**126 (69%)**	**51 (61%)**
**Pain/discomfort**	**8 (4%)**	**4 (5%)**	**111 (60%)**	**48 (59%)**	**65 (35%)**	**30 (37%)**
Anxiety/Depression	**82 (45%)**	**30(37%)**	**57 (31%)**	**37 (46%)**	**43 (24%)**	**14 (17%)**

The overall quality of life weight for the worst day was 0.29 for the confirmed cases and 0.34 for the ILI controls (Table 4). After the symptoms had gone the quality of life weights were 0.96 and 0.97 respectively. Based on the VAS scale the QALY weight was 90 (on scale 0–100) for the background and 30 for the worst day. The comparable values for the ILI controls were similar, 89 and 30 respectively. Complete information to calculate an overall QALY loss was only available for 114 of the 186 (61%) confirmed cases and 46 (55%) of the 83 control ILI cases. The final QALY loss due to the whole period of disease was 0.008 for the confirmed cases and 0.0075 for the cases in the control group, i.e. 2.9 and 2.7 Quality Adjusted Life Days (QALDs), respectively.

**Table 4:** Impact of ILI on health related quality of life for H1N1v confirmed and non-H1N1v ILI control patients.

	*Confirmed H1N1v ILI cases*	*ILI controls (non-H1N1v cases)*
EQ-5D Background (min-max,median)	0.96 (0.15–1,1)	0.97 (0.5–1,1)
EQ-5D Worst day (min-max,median)	0.29 (−0.073–1,0.24)	0.34 (−0.073–1,0.24)
VAS Background (min-max,median)	90 (20–100,95)	89 (55–100, 90)
VAS Worst day (min-max,median)	30 (0–100,25)	30 (5–80,30)
Overall QALY loss (min-max,median)	0.008 (0–0.027,0.006)	0.0075 (0–0.044,0.006)
Overall QALD loss (min-max,median)	2.92 (0–9.84, 2.18)	2.74 (0–16.2, 2.12)

In the multivariable linear regression only antiviral use (within 48 hours) was associated with the number of QALDs lost, and only in confirmed H1N1v cases (p = 0.084). Prompt antiviral use was found to reduce the number of QALDs lost by 50% (22%–110% CI 95%). No other factor (including age, sex, presence of risk-factors, whether hospitalised, whether the case was confirmed H1N1v or not) was significantly associated with the number of QALDs lost.

### Systematic literature review

Sixty-one articles were found, 10 of which were reviews and discarded. A further 10 studies only estimated life years lost, two papers described different diseases, a further two were not published in English, leaving 36 studies mentioning the burden of influenza or ILI. However, none of the reviewed papers was specifically dedicated to the burden of disease, but gave values for this as part of a cost-effectiveness study. A number of papers present the same data from the clinical trials of the antiviral zanamivir but with different analyses. Overall, we were only able to identify four original sources of information on the burden of disease due to ILI as measured in QALYs, including the trial data as one source, see table 5 for an overview.

**Table 5:** Overview of different published estimates of QALDs lost due to ILI, for sources which presented data or interpretation of that data.

*Study*	*QALY weight Worst day/disease*	*Background QALY weight*	*Duration of disease (days)*	*QALD loss*	*Sample size*	*Method*	*Focus group*	*Age group*
(Griffin, Perry, & Fleming, 2001)	−0.066	0.817	2.48	2.19*	21	EQ-5D	Patients (within 3 months after onset)	18+
(Griffin, Perry, & Fleming, 2001)	−0.263	0.72	2.48	2.45*	8	EQ-5D	GPs	Unknown
(O'Brien, Goeree, Blackhouse, Smieja, & Loeb, 2003)	Changed by day	Not reported	Not reported	Not reported	920 in placebo (639 with influenza	Likert score transferred to VAS	ILI patients (clinical trails antivirals GSK)	18–64
(Turner et al., 2003)	Changed by day	Not reported	Not reported	Not reported	See O'Brien at al	Converted the Likert scores into VAS scores converted those into time-trade off scores	ILI patients (clinical trails antivirals GSK)	18–64
(Rothberg, Bellantonio, & Rose, 2003)	0.25	1	Not reported	Not reported	15	HUI-3	Patients	Working adults
(Prosser et al., 2006)	Not reported	Not reported	Not reported	1.83	Not reported	Time trade off	Parents of children	Children
(Siddiqui & Edmunds, 2008)	VAS scores as presented by O'Brien et.al	0.85	Not reported	1.68 for influenza ILI1.57 for non-influenza ILI	See O'Brien et.al.	VAS scores substracted from the baseline	ILI patients	18–64
(Sander et al., 2010)	QALY scores as presented by Turner et.al.	Not reported	Not reported	5.33 (0–19 yrs)6.35 (20–64 yrs) 10.69 (65+)	See O'Brien et.al	Uses published QALY weights and combines this with unpublished disease duration	ILI patients	18–64

The first original source of data is a study by Griffin et al. [10] in which 21 working adults were asked to fill an EQ-5D questionnaire within 3 months of onset of ILI, and 8 GPs were asked to do the same. The study reported relatively low QALY weights for ILI with values below zero (corresponding to a state worse than death) being recorded. The weights were, however, applied to a very short duration of illness which was measured separately on a different group of patients (2.48 days). Hence the overall loss was estimated at 2.19 QALDs. The second source of data is the clinical trials of zanamivir, reported by O'Brien et al. [11] In the zanamivir trials almost 640 patients with ILI were asked within 48 hrs of onset of disease to value their health on a scale between 0 and 10 every day for 21 days. Since this is not a QALY scale, several separate analyses have been performed on the same data to map the disease-specific scale onto a QALY scale. In addition, since these data have mostly been used in cost-effectiveness studies of the use of antivirals, no figures for overall QALY loss due to ILI have been published, only the difference in QALY loss due to ILI in patients with and without antivirals [11]–[14]. Only two studies [15], [16] use these data to estimate the overall QALY loss: the first uses a separate estimation of the background quality of life weight based on population estimates and the second a separate estimation of the duration of illness. The final estimates differ by up to 6-fold. The QALD lost estimated by Siddiqui et al. [16] is 1.68 for complicated influenza and 1.57 for non-complicated, non-influenza ILI whereas the QALD loss calculated by Sander et al. [15] is 5.33 for 0–19 yrs, 6.35 for 20–64 yrs and 10.69 in over 65 s. A third potential source of QALY loss data is a study in which 15 randomly selected working age patients and health care workers [17] were asked to fill in the HUI-3 questionnaire based on their recollection of their most recent episode of ILI. The results were used to estimate a quality of life weight of 0.25 for an individual with ILI. Unfortunately, the duration that someone is in this state was not determined and so no QALY loss due to an episode of ILI can be easily calculated from these data. The fourth source of data is a study by Prosser et al. [18] in which parents were asked how much time they were willing to trade off their own life to prevent ILI in their children, which resulted in a value of 1.825 QALDs lost per ILI case.

### Total burden of disease pandemic

Given the estimated QALY loss in this study the overall burden of the 2009 H1N1v pandemic in England was around 28,126 QALYs (22,267 discounted). This is because almost a 7.8 million people were estimated to be infected with the novel virus over the course of the two waves of disease. Of these, around 2.1 million were estimated to have experienced fever and there were 337 deaths [5]. Mortality accounted for 40% (24% with discounting) of the QALY loss attributable to H1N1v.

## Discussion

Given the global public health significance of influenza, it is remarkable how few studies have tried to quantify the morbidity and mortality impact in QALYs of this ubiquitous disease. In addition, as our systematic review reveals, the studies that have been performed often have considerable methodological limitations. For instance, two of the studies were small and retrospective [10], [17], two studies collected data from proxies (such as GPs) in addition to or instead of patients [10], [19], and the studies based on the zanamivir trial did not use standardised instrument and only estimated the difference in QALYs lost when on antivirals [11]. Finally, many of the studies did not estimate the duration of illness, and no previous study explicitly mentions their assumptions about the shape of the QALY loss (e.g. rectangular or triangular). This study is the only prospective population-based study of the health-relative quality of life impact of confirmed influenza and influenza-like illness that uses a standardised and well-validated instrument (the EQ-5D). The study shows that the overall QALY loss for confirmed H1N1v and other non-H1N1v influenza-like-illness was similar, at around 2.8 QALDs per patient. The study also confirmed that the range of symptoms and the severity of illness appeared similar in the two groups of patients, with the vast majority of patients reporting some problems with usual activities and pain and discomfort when they were ill with influenza or ILI. Only the prompt use of antivirals was significantly associated with a reduction in the QALDs lost, and only in the confirmed cases. Although deaths from H1N1v were comparatively rare, our study suggests that the overall burden of illness was considerable with more than 28,000 QALYs lost over the two waves of infection in England. This compares with an estimated QALY loss per year of 18,000 for chickenpox and shingles combined [20] and 97,000 for type 1 diabetes [21]. However compared to a high mortality disease such as coronary heart disease which has an estimated annual burden of 8.2 million QALYs lost [21], it is relatively small.

The main strength of the study was that it was a population-based prospective controlled study. The study was carried out during a period when every case seeking health care actively was investigated, with follow-up of all confirmed cases and their contacts. Therefore, it should be as representative a study is likely to be possible. Indeed, during the period of the study the two regions that were most heavily affected at the beginning of the epidemic (London and the West Midlands) stopped investigating every possible case, and so we excluded data from these regions to prevent bias. Nevertheless the possibility remains that more severe cases were more likely to come to the attention of medical authorities. In addition, although the overall response rate was good for a postal survey (>40%), there is always the possibility that more severely affected patients were more likely to return the questionnaire. Most patients probably knew their status (i.e. whether they were a confirmed H1N1v case or not), which may have led to the differential response between confirmed and other ILI cases. Hence, although every effort was made to reduce bias, there remains the possibility that the average loss estimated here is an overestimate of the true QALY loss per case.

The total burden of influenza in the population is probably underestimated, however, as we do not include the QALY loss from afebrile cases i.e. those without fever. Only patients with ILI were investigated and their data recorded on the Fluzone database. Thus patients with milder symptoms – particularly those lacking fever – were not followed up. Carat et al. [22] suggest that about one half of influenza patients with respiratory symptoms do not develop fever. These individuals probably have a lower QALY loss than febrile cases. Indeed, of the 18 individuals who reported not having fever, 7 responded to both questionnaires, with an average loss of 1.2 QALDs per case. Little weight should be put on these numbers as the study was not designed to ascertain the burden of non-febrile acute respiratory illness, and the sample is small. However, as there may have been large numbers of patients without fever their contribution to the overall burden may have been significant. A preliminary literature review for QALY loss for acute respiratory illness revealed no papers, and so this remains an area for further study.

Our findings suggest that prompt use of antivirals reduces the number of QALDs lost. There are (to our knowledge) no other data on the effect of antivirals on health related quality of life of H1N1v patients. Our findings confirm the results from clinical trials on seasonal influenza [11], and are also in accordance with virological data, which seem to suggest that antivirals reduce viral load in H1N1v infected patients [23]. Other factors, such as age, were not significantly associated with severity as measured by QALDs lost, which also seemed to confirm the findings of virological studies of H1N1v [23].

This study provides important baseline information on the severity of H1N1v and other influenza-like-illnesses that can be used to judge the overall impact of these diseases on the health of populations. This will facilitate rational decision-making regarding the control of influenza over the coming seasons.
